# Ulcerations of the Gums and Exfoliation of the Alveolar Processes of Children—Resulting from General Debility or Defective Nutrition

**Published:** 1843-12

**Authors:** C. A. Harris

**Affiliations:** Surgeon Dentist, Baltimore.


### ARTICLE III.

*Ulcerations of the Gums and Exfoliation of the Alveolar Processes of children—resulting from general debility or defective nutrition.* By PROF. C. A. HARRIS, M. D. Surgeon Dentist, Baltimore.

THE gums and alveolar processes of children are occasionally attacked by a very peculiar and singular form of disease. Several cases of which, having come to my notice, I have thought that a description of it might not be uninteresting to the medical reader, and especially as it is a disease, that, if I am correctly informed, has never been noticed by an English or American medical writer. It has, however, been very accurately described by several

French authors,—particularly by DELABARRE, whose opportunities for observing it, in every variety of form, seem to have been very ample. The description given of it by him, accords so well with that of the cases which I have seen, that I shall quote what he says respecting it.

"There is a disease that is very rare among the wealthy, but very common among the indigent. It especially manifests itself about the period of the moulting of the temporary teeth. It is frequently observed on children that appear to be healthy, as well as on those of scrofulous or scorbutic affections.

"Can it be a peculiar disease still unknown? M. DUVAL has already noticed this disposition: and among the great number of children that are continually brought to the orphan asylum, I have frequent occasion to observe singular complications of this affection, which are modified according to the differences of the strength, sex, and idiosyncrasies of the different subjects. In some, the lips and gums are of a beautiful, bright red color; in others the lips are rosy, though the gums are pale; or both of them are reddish, and much swollen. Sometimes also the gums are pale and the lips of a deep red, there is besides no scorbutic or scrofulous symptoms; frequently the child is robust and appears to be quite well.

"It begins to experience troublesome itchings in the gums; they ulcerate around the necks of the temporary teeth, which become separated from them, and somewhat loosened. If the symptoms continue, the evil is propagated, a burning pain is felt in some parts of the buccal membrane that lines the cheeks; and ulcerations of a greater or less size are formed there. To these evils are joined some others—painful swellings in the glands that environ the lower jaw, the entire separation from the gum, and loss of undecayed teeth, whose roots are frequently surrounded with a yellowish mucous tartar. I have even known portions of the alveoli to exfoliate, and then the cure is effected almost naturally. Whatever may have been the importance and continuance of the local maladies, I have observed that, if children reach the seventh or eighth year, their second teeth are not deranged by the cause that occasioned the loss of the first; they are only badly arranged by reason of the want of development in the maxillary bone, which shows that during the disease the general ossification is carried on but slowly."

The symptoms as here described by M. DELABARRE, correspond for the most part with those of the cases which I have seen, except that they present a somewhat greater variety; the color of the gums and lips in the cases that have come to my notice, were always that of a deep red, sometimes bordering on purple. The separation of the gums from the alveolar processes, was, in every instance, preceded by ulceration of their edges. The matter formed, was at first, muco-purulent, but afterwards become ichorous, and increased the irritation of the parts with which it came in contact. As the gums separated from the sockets of the teeth, it denuded them of their periostea, and insinuated itself into the alveolar cavities and caused the destruction of their membranes.

The disease having arrived at this stage, necrosis and exfoliation of the alveolar processes soon followed—carrying away with them the deciduous, and sometimes the embryo adult teeth. In one instance four of the second teeth that had come through the gums were removed with their exfoliated sockets.

As it regards the effects produced upon the permanent teeth by this affection, they were in the few cases that have fallen under my notice, decidedly injurious. In some instances their enamels were covered with dark spots; in others they were corroded, and when the disease had occurred early in life, their bony structure appeared soft and chalky, and their liability to be acted on by chemical agents greatly increased. My observations, therefore, on this subject differ somewhat from those of DELABARRE'S. He tells us that, "frequently the child is robust and appears quite well." In all the cases which I have seen, the general health was evidently affected, and in some very considerably. Usually, the pulse was small and quick, the skin dry and husky, the bowels constipated, much lassitude of body, and great disposition to sleep. These symptoms, however, I have never known to precede the local affection, but seem rather to have been symptomatic of it.

Continuing the subject, M. DELABARRE says, "Chilblains of a bad character are the portion of these children; their skin is terreous or wan, the flesh of some is soft, their eyes dull, their habit of body languid, and finally have true scrofulous obstructions

Believing that I have, in these cases, found that the general weakness depends on that of the digestive organs, whence result divers degrees of alterations in the vivifying properties of the fluids, I employ with success, bitters and tonic powders, such as Jesuits' bark, the small centaury, &c."

I would here just remark, that I have never met with an instance of this disease in which there was not evidently a scorbutic tendency of the general system, and am disposed to believe, from the very nature of the affection, that, although there may exist in some cases a scrofulous diathesis, that this always predominates.

"For the local treatment of the gums," says our author, "I recommend acidulated gargles, or those in which have been dissolved some grains of sulphate of zinc. If these means are not sufficient, I have the ulcerations cauterized every day with dissolved *argentum nitratum.* Many may be cured by these simple remedies; but I have sometimes been obliged to have recourse to the actual cautery, from which I have derived great advantage. Several, however, in whom the disease was very violent, have sunk under it, after having experienced the following symptoms:

"General swelling of the stomatic membrane, ulceration on one side alone, prostration of strength, slight pulse, redness of the affected side, avidity for food, burning thirst, then, at the expiration of three or four days, a gangrenous spot, resembling an anthrax either below the cheek bone or about the lips; rapid increase of the spot, which at first livid, becomes black the same day, and, when grown to the size of a five franc piece, separates in the middle, and forms an obstinate ichorous ulcer, whose pale edges roll upon themselves like flesh exposed to the action of a very brisk fire. The whole of one side of the face is soon entirely devoured; the bones are uncovered as well as the roots of the teeth, which, however, are not carious but fall out of themselves; a hectic fever consumes these unhappy beings, to whom death is too tardy a relief. This sad scene has passed before my eyes in the cases of two children, one of them eight, and the other a girl of nine years old. Death seizes them between fifteen and twenty days after the attack of the first marked symptoms, notwithstanding all the rational means employed, such as tonics and caustics. These are the characteristics, which an inveterate cancerous affection, with all its horrible scent, would not exhibit. This affection has been so well described by M. BARON, that we cannot but profit by his reflections and try the means recommended to be employed by him in similar cases.*

"In general, the female sex is more subject to affections of this nature than the male. The dissections that I have made of several children, attacked with this disease, and in different stages of it, have always presented me the bones singularly softened. Those of the jaw, especially, are very easily cut with a scalpel. There is consequently but a very small quantity of calcareous phosphate in them, but their teeth do not appear to be the less hard on that account; on pressing the cutting edges of these, which have shot up, we perceive them bend, and, as it were, sink again into the jaws.

"Some authors, it seems to me, have referred this kind of disease to a sort of scrofula; but, for reasons, which I find myself compelled to detail, I do not believe this opinion can be adopted, because the seat of this last affection is in the lymphatic, whence it may be propagated to different systems, while the affection, the symptoms of which I have just described, has its seat in the *organs of nutrition, and, in the fluids that are conveyed to them.* Moreover in the beginning of the disease, there are no obstructions that can reasonably be regarded as of a scrofulous nature, but such may occur as it gradually progresses and propagates the disturbance in all the secretions.

"This vicious disposition, which I have frequent opportunities of observing in the asylum to which I am attached, is sometimes innate in children, and sometimes acquired by their suffering great privations of nourishment. In the former case they are melancholy, and grow for a long time without complaining of any pain; both their dentitions are very tardy, the bones of the jaws as well as those of all parts of the body, are exceedingly slow in their development, the membrane of the lips and mouth is pale, the saliva mucous; these children are meagre, their skin soft, which has led me to adopt the word *astheny,* to denote this state, for it designates the want of strength that exists in all the tissues of these little beings, whose nutrition is effected with extreme difficulty, with-

* Vide le Bulletin de l'Ecole de Medecine de Paris, 1816, page 136.

out there being any possibility of attributing the cause of it to a distinct disturbance of any organ, separately considered.

"There appears, therefore, a want of vitality in all the apparels which causes the child to waste away daily, and the natural functions to be performed with a sort of supineness. It has no appetite, is frequently constipated; at other times has diarrhœa, but no fever; it is timorous and nervous, has a melancholy air, and, in short, excites a sentiment of compassion. It seems as if sanguification could not be effected; for in these subjects the blood is very serous and almost colorless. After what has been said, it is not surprising, that the organic exhalents of the bones should not supply them with scarcely any calcareous earth, but with a material, that is without consistence, acidulated and rather destructive, than reparative.

"Exercise in the open air, generous wines, sometimes even a little alcoholic liquor, and a diet not exuberant, but consisting of succulent viands, form a part of the regimen, which, in these cases, it is indispensable to follow. It is necessary to prohibit milk and acid or aqueous aliments. To arouse the vitality, I have constantly and successfully employed, the *juice of cruciferous plants, the guinea in powders*, but with this last medicine I think useful to unite opium, which diminishes its action on the digestive organs.

"I banish the use of ptysans, which generally fatigue the stomach: vescatories and purgatives are employed, but with much circumspection, and only when there is some reason for displacing an irritation communicated to some interior organ."

Having now quoted what M. Delabarre says respecting this most singular affection, little remains to be said by me on the subject. In conclusion, however, I would observe, that none of the cases in which I have been consulted were of so aggravated a character as some of those which he has described. Except in three instances, in which exfoliation of the sockets of several of the teeth had commenced previously to my seeing the patients, the disease yielded to astringent, detergent and acidulated gargles, the application of *nitrate of silver*, in strong solution, to the ulcerated edges of the gums, aperients, tonics and a generous nutritive diet. The others were cured soon after the completion of the exfoliation of the dead sockets, by similar treatment and the restorative operations of the economy.

From the cases which have fallen under my observation, I have been induced to adopt the opinion advanced by the author whose remarks on the subject I have quoted, that the disease is the result of general debility, occasioned by privation or a want of proper nourishment, for its occurrence, so far as my knowledge extends, is always confined to the indigent. I am, however, at the same time, inclined to the belief, that it is favored by a constitutional tendency of the general system, though, notwithstanding Delabarre seems to think otherwise, and that without this, other causes would be insufficient to produce it.

To the ordinary spungy and inflamed gums, or scurvy, or conjoined suppuration, as the affection is sometimes called, the disease in question bears but little analogy. That affection is the result of local irritation, favored, it is true, in many instances, by a general scorbutic diathesis, produced by salivary calculus, decayed or dead teeth, or roots, or a vitiated state of the buccal secretions, and its progress, compared with this, is very slow. In that disease, the matter that is formed by the suppuration of the gums is, in most instances, healthy pus, but in this, although at first it is muco-purulent, it soon becomes ichorous and intolerably offensive. The sockets of the teeth too, by that affection, are never suddenly laid bare, but by this they are often in a very few days denuded of the gums, deprived of vitality and caused to exfoliate. To the attacks also of that disease, all ages, classes, and conditions of people are liable, whereas, to those of this, the indigent alone are subject, and these only during the earlier periods of life.

*Maryland Med. and Surg. Jour.*

